# Inherited salt retention is associated with increased IL-17 responses and autoimmunity.

**DOI:** 10.1172/jci.insight.206932

**Published:** 2026-07-16

**Authors:** Muhammad Atif Rauf, Sanskriti Agarwal, Rebecca R. Baker, Jennifer Steeden, Alfredo Petrosino, Maria Kiliaris, Robert Unwin, Keith Siew, Alan D. Salama, Rhys D.R. Evans

**Affiliations:** 1 Centre for Kidney and Bladder Health and; 2 UCL Centre for Translational Cardiovascular Imaging, University College London, London, United Kingdom.

**Keywords:** Immunology, Nephrology, Adaptive immunity, Autoimmune diseases, Sodium channels

## Abstract

Patients with Familial Hyperkalaemic Hypertension (inherited salt retention) have increased sodium stores, dysregulated immunity, and are more prone to developing autoimmune disease.

**To the Editor:** Autoimmunity is increasing, with approximately 1 in 10 people affected in the United Kingdom (1). Environmental triggers have been proposed to explain this, including increased dietary salt (sodium chloride) (2). Sodium activates proinflammatory Th17 cells (3), and we have previously shown that inherited salt-losing tubulopathies (SLTs) are associated with immunodeficiency due to impaired IL-17 responses (4). Immunological consequences of chronic sodium excess, however, are unknown. Here, we investigate immunity in patients with familial hyperkalemic hypertension (FHHt; Gordon’s syndrome), an ultrarare inherited salt-retaining tubulopathy due to mutations causing constitutive activation of the sodium-chloride cotransporter (NCC) in the kidney (5).

Thirteen patients with genetically confirmed FHHt were included. Mutations in *KLHL3*, *WNK4*, and *WNK1* were present in 9 (69%), 3 (23%), and 1 (8%) patients, respectively. Demographic, clinical, and biochemical features of patients with FHHt at presentation and recruitment are outlined in Supplemental Table 1 and Supplemental Figure 1 (supplemental material available online with this article; https://doi.org/10.1172/jci.insight.206932DS1). Increased tissue sodium in FHHt was confirmed with lower limb ^23^Na MRI, with apparent sodium concentrations similar to those reported in other sodium excess states (6) (Figure 1, A and B; Supplemental Table 1; and Supplemental Figure 1).

Clinical features of immune dysregulation were determined by focused history and compared with those of patients with SLT and healthy individuals and patients with tubular disease without a primary sodium handling defect acting as controls. Patients with FHHt had increased viral infections, specifically recurrent human papilloma and herpes simplex virus, compared with all control groups (Supplemental Table 2). Moreover, 31% of patients with FHHt had autoimmune disease (patients with Crohn’s disease = 2, patients with juvenile idiopathic arthritis = 1, patients with immunoglobulin A nephropathy = 1), significantly more than recent estimates in the general population (*P* = 0.036) (1).

Initial immunological investigation demonstrated an increased CD4/CD8 ratio in 4 (31%) patients with FHHt (Supplemental Table 3 and Supplemental Figure 2). Based on the evidence linking sodium to Th17 (CD4^+^IL-17^+^) polarization and our work in SLTs, we hypothesized that FHHt would show enhanced Th17 responses. To explore CD4^+^ T cell subsets, we stimulated PBMCs with anti-CD3 and anti-CD28 for 3 or 7 days with optimal Th17 polarizing conditions and determined Th1 (CD4^+^IFN-γ^+^) and Th17 populations by flow cytometry. Th1 responses were reduced in patients with FHHt compared with those in individuals acting as healthy controls after 3 days (Figure 1, C and D), consistent with their increase in viral infections. IL-17 responses were increased at 3 and 7 days, with an increase in Th17 polarization, the ratio of Th17/Th1 cells, and secreted IL-17 (Figure 1, E–G), in keeping with the propensity of patients with FHHt to develop autoimmunity.

We proposed that exposure to increased tissue sodium causes immune dysregulation in FHHt. To test this, we stimulated PBMCs from individuals acting as controls under Th17 polarizing conditions with additional sodium. Adding sodium to culture conditions increased IL-17 responses and attenuated IFN-γ responses (Figure 1H), mimicking the FHHt immunophenotype.

Furthermore, we assessed immune responses in patients with FHHt treated with thiazide diuretics, NCC inhibitors that deplete sodium. Thiazide-treated patients trended toward reduced immune dysregulation (Figure 1I). Given NCC expression in various immune cells (4), we investigated whether this was due to a direct effect of thiazide diuretics on T cells. We assessed IL-17 responses from patients with FHHt and individuals acting as healthy controls in the presence of hydrochlorothiazide, confirming no direct effect (Figure 1J).

Therefore, we hypothesize that thiazide treatment indirectly mitigates immune dysregulation in FHHt by inhibiting NCC and depleting body sodium. Supporting this, we correlated immunophenotype to readouts of NCC activity (fractional excretion of chloride [FeCl] and urinary calcium/creatinine ratio [uCa/Cr]). As expected with NCC inhibition, thiazides reduced uCa/Cr and increased FeCl. IL-17 responses correlated positively with NCC activity (negatively with FeCl), whereas IFN-γ responses correlated negatively with NCC activity (negatively with uCa/Cr) (Figure 1, K and L).

Therefore, we show that inherited salt retention is associated with clinical features of dysregulated immunity, including increased viral infections and autoimmunity. These features are associated with increased IL-17 and decreased IFN-γ responses, the opposite of findings in SLTs (4). We propose that altered sodium balance in FHHt creates tissue environments with increased sodium concentration, which may include sites of inflammation and lymphatics. Resultant T cell exposure to higher sodium contributes to immune alterations, and diuretics may attenuate these effects, analogous to sodium depletion in SLTs.

Our findings provide further evidence for a role of sodium balance in immunity. Future work should examine the direct effects of FHHt-associated mutations on T cell signaling, and whether other states of chronic sodium retention (e.g., primary aldosteronism) have comparable immune features. Prospective studies are needed to confirm thiazide-mediated NCC inhibition reduces tissue sodium in FHHt and if alterations of sodium balance can serve as adjunctive immunomodulatory treatments.

For detailed methods, information regarding sex as a biological variable, statistics, study approval, data availability, author contributions, and acknowledgments, see the supplemental materials.

## Funding support

- Kidney Research UK Training Fellowship (TF_007_2016112) to RE.
- UK Research and Innovation Fellowship (MR/S032290/1) to JS and RRB.

## Conflict of interest

The authors have declared that no conflict of interest exists.

## Supplementary Material

Supplemental data

Supporting data values

## Figures and Tables

**Figure 1:**
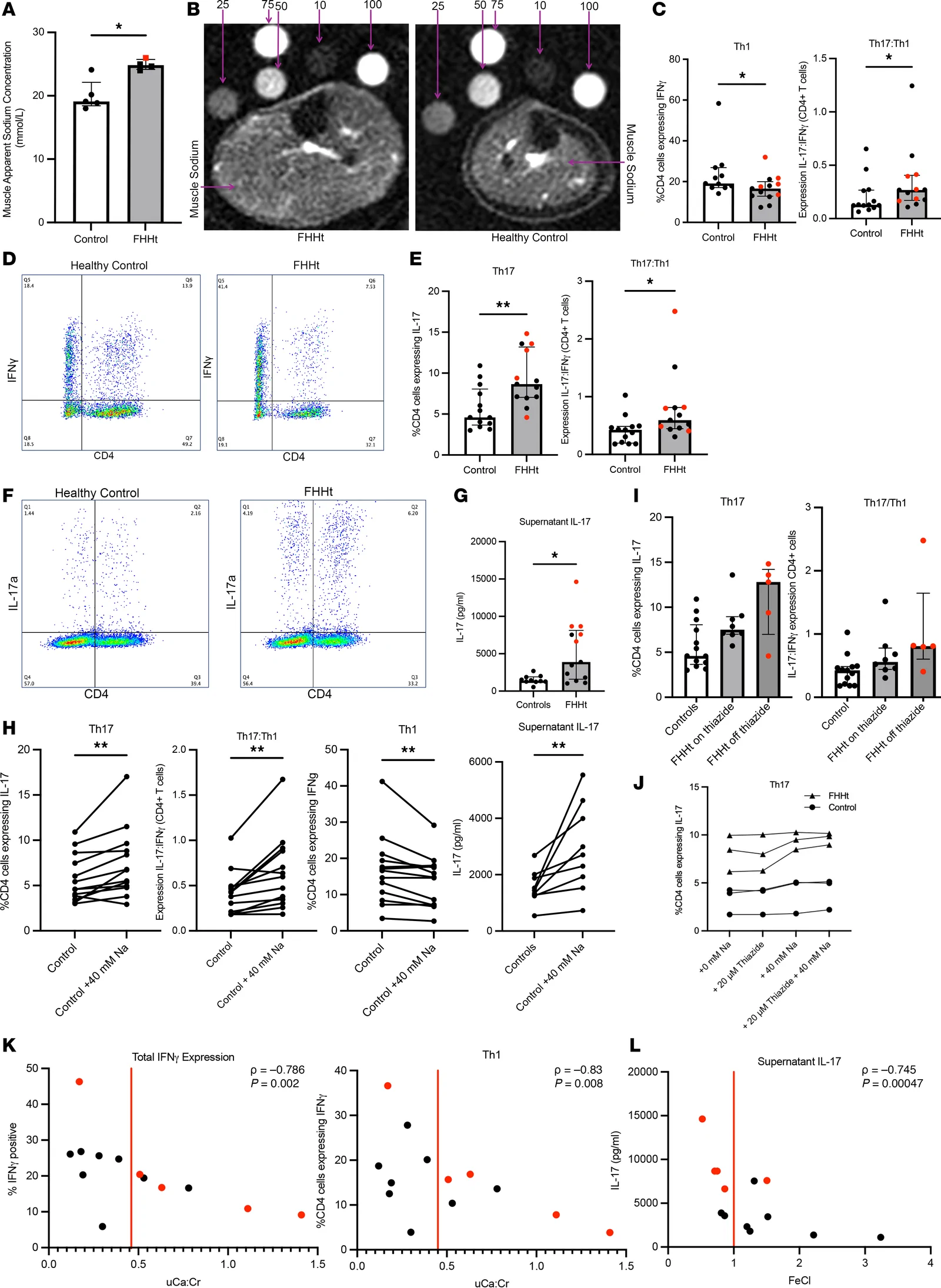
Sodium storage and immunological investigation of FHHt patients. (**A**) Calf muscle apparent tissue sodium (^23^Na MRI) in FHHt (*n* = 84) and individuals acting as healthy controls (HCs, *n* = 85). (**B**) Representative ^23^Na MRI images; calibration phantoms (10–100 mM NaCl) are labeled. (**C**) Th1 polarization and Th17/Th1 ratio (3-day PBMC stimulation), with representative FACS plots (**D**). (**E**) Th17 polarization and Th17/Th1 ratio as well as (**G**) supernatant IL-17 concentration (7-day stimulation), with representative FACS plots (**F**) in FHHt and HCs. (**H**) Th17 and Th1 polarization, Th17/Th1 ratio, and supernatant IL-17 concentration in HC cells cultured with and without +40 mM NaCl. (**I**) Th17 polarization and Th17/Th1 ratio in patients with FHHt on (*n* = 8) and off (*n* = 8) thiazide. (**J**) Th17 polarization in patients with FHHt and HCs (*n* = 8) with hydrochlorothiazide (20 μM), NaCl (40 mM), or both (7-day stimulation). (**K**) IFN-γ expression and Th1 polarization vs. urinary calcium/creatinine. (**L**) IL-17 vs. fractional excretion of chloride in FHHt. Red lines represent the upper limit of normal. Dots represent individuals. Red symbols denote patients with FHHt off thiazide. Groups in **A**, **C**, **E**, and **G** were compared by 2-tailed Mann-Whitney test; in **H** by paired Wilcoxon test; and in **K** and **L** by Spearman’s correlation. Bars show the median and IQR. *P < 0.05, **P < 0.01. Data in **B**, **D**, and **F** represent 9, 26, and 26 experiments, respectively.

## Version 1 07/17/2026

In-Press Preview

## Version 2 08/24/2026

Electronic publication
