# Supplementary material for: Inherited salt retention is associated with increased IL-17 responses and autoimmunity
Source: JCI Insight. 2026 Jul 16;11(16):e206932. doi: 10.1172/jci.insight.206932 (PMC13502187; doi:10.1172/jci.insight.206932)

Materials and methods

FHHt and control cohorts

Patients with genetically confirmed FHHt were recruited from the Tubular Kidney Diseases Clinic at the Royal Free Hospital, London, UK. Demographic, genetic, biochemical, and clinical data were recorded at the time of recruitment and documented from the time of presentation to kidney services. Patients with genetically confirmed salt-losing tubulopathies (SLTs) (Bartter syndrome = 23, Gitelman syndrome = 22, and EAST syndrome = 2) and disease controls (patients with tubular disease without a primary sodium handling defect - proximal tubulopathy = 7, genetic distal Renal Tubular Acidosis (dRTA) = 3, tubulointerstitial nephritis (TIN) = 3, hypokalaemia due to eating disorder = 2, STX16 mutation = 2, TRPM6 Magnesium wasting = 1, Medullary Sponge Kidney = 1) were recruited from the same clinic. Healthy controls were age- and sex-matched volunteers who had no known medical conditions.

Individuals underwent a structured clinical history focused on clinical features of dysregulated immunity, including infectious, atopic, allergic, and autoimmune disease, which were recorded in a protocolised manner. Clinical variables and prevalence of immune-mediated diseases were compared between FHHt patients and control groups.

Sex as a biological variable

Both male and female patients with FHHt were recruited, and control groups were sex matched.

^{23}Na MRI Imaging

To determine muscle sodium storage, FHHt patients (n=4) and HCs (n=5) underwent sodium (^{23}Na) MRI imaging of the lower limb. Participants were scanned on a 3 T mMR Biograph with a ^{23}Na transmit/receive birdcage coil (Stark Contrast MRI Coils Research). Calibration phantoms with nominal ^{23}Na concentrations of 10, 25, 50, 75, and 100 mM were placed on the participant's limb within the field of view (FOV). ^{23}Na images were acquired using a previously described 2D ultra-short-echo spiral sequence (Baker et al., *MRM,* 2023) with the following parameters: FOV = 180 × 180

mm^2, pixel size = 2.25 × 2.25 mm^2, slice thickness = 30 mm, TR = 100 ms, TE = 0.23 ms, flip angle = 90°, averages = 150, and acquisition time = 10:15 min:s. Hydrogen (^1H) gradient echo images were also acquired with the same FOV and slice thickness, but higher spatial resolution for segmentation of the structures (pixel size = 0.35 × 0.35 mm^2, TR = 8.6 ms, TE = 4.0 ms, flip angle = 20°, acquisition time = 2 s).

Quantitative ^{23}Na maps were calculated by drawing a region of interest in the center of each phantom and performing a linear calibration. Regions of interest were drawn in the skin and muscle on ^1H images and transferred to ^{23}Na maps to determine mean apparent tissue sodium concentration (aTSC) in the tissues. All analysis was performed using the Horos viewing platform (Horos 4.0.1). Mean aTSC in the skin and muscle were compared between FHHt patients and healthy controls.

Biochemical and Initial Immunological analyses

Biochemical and initial immunological analyses were undertaken for patients in the National Health Service (NHS) clinical laboratories at the Royal Free Hospital. Immunological analyses included measurement of full blood count, C-reactive protein (CRP), immunoglobulin A, G, M, and E concentrations, complement protein C3 and C4 concentrations, lymphocyte counts and subsets (CD3+, CD4+, CD8+, CD19+, CD16/56+), and antinuclear antibody levels.

3-day PBMC stimulation

Subsequent investigation was undertaken in research laboratories. To assess T cell responses, peripheral blood mononuclear cells (PBMCs) were isolated from FHHt patients (n=13) and HCs (n=13) by density centrifugation (Lymphoprep; Stemcell, cat#07861). Cells were plated into a 96-well round bottom plate at a concentration of 4 million cells per ml and stimulated for 72 hours with anti-human CD3 (10ug/ml plate bound; BD Pharmigen, cat# 555336) and anti-human CD28 (1ug/ml; Invitrogen, cat# 16-0289-85) in XVIVO15 media (Lonza, cat# BE02-060F). On day 3, cells were restimulated for 4 hours with PMA (50ng/ml) and ionomycin (1ug/ml; Sigma, cat#I9657) in the presence of brefeldin A (5ug/ml) for intracellular cytokine staining.

7-day PBMC stimulation

Further analysis of IL-17 responses was undertaken by stimulating PBMCs from FHHt patients (n= 13) and HCs (n= 13) with anti-human CD3 (10ug/ml plate bound), anti-human CD28 (1ug/ml), IL-1β (12.5ng/ml; Peprotech, cat#200-01B), IL-6 (25ng/ml; Peprotech, cat#200-06), IL-21 (25ng/ml; Peprotech, cat#200-21), IL-23 (25ng/ml; Peprotech, cat#200-23), and TGFβ (5ng/ml; Peprotech, cat#100-36E) in XVIVO15 media (measured sodium concentration 131mM) for 7 days. Experiments were also undertaken with cells from HCs (n=13) in the presence of additional NaCl (+40mM), and in patients with FHHt (n=3) and HCs (n=3) in the presence of the NCC inhibitor hydrochlorothiazide (20uM; Sigma, cat#H4759). On day 7, cells were restimulated for 4 hours with PMA (50ng/ml) and ionomycin (1ug/ml) in the presence of brefeldin A (5ug/ml) for intracellular cytokine staining.

An additional sodium concentration of 40 mmol/L was selected as we have previously demonstrated that this provides the maximal impact on IL-17 responses without affecting cell viability; moreover, it facilitates comparison with prior work. ^{23}Na-MRI captures *apparent* tissue sodium and likely underestimates free-sodium concentrations at sites of T cell activation. The 'high sodium' concentration (171mM) used *in vitro* in this study is reflective of *in vivo* sodium concentrations previously measured at interstitial sites (Machnik et al. Nat Med 2009; Jantsch et al. Cell Metab 2015; Jobin et al. Sci Transl Med 2020).

Flow Cytometry

After restimulation, cells were stained for viability (Fixable viability dye – efluor450; Invitrogen, cat# 65-0863-14), CD4 (CD4-FITC; BioLegend, cat# 300538), IL-17 (IL-17-PE; BioLegend, cat#512306), and IFNγ (IFNγ-APC; BioLegend, cat#502512). The proportions of viable CD4+ cells expressing IL-17 (Th17) and IFNγ (Th1) were determined by flow cytometry on a BD LSR Fortessa flow cytometer. Flow cytometry analysis was carried out on FlowJo v10. Gating for flow cytometry experiments was via fluorescence minus one and gating strategies are outlined in **Supplementary 4**.

ELISA

On the final day of stimulation for 3 and 7 day protocols, supernatants were collected and stored frozen for subsequent measurement of IL-17 concentrations by ELISA (R&D systems, cat#DY317).

All assays were performed contemporaneously with matched healthy controls; absolute frequencies are not directly comparable to those of our previous study (Evans et al. 2020) due to differences in cohort, reagents, and instrument calibration.

Statistics

Data are presented as number and percentages for categorical variables, and median and interquartile range for numerical variables. Categorical variables were compared using the Fisher's exact or Chi-squared test. Numerical variables were compared between two groups using the Mann-Whitney test and the Wilcoxon test (both two-tailed) for unpaired and paired data respectively. Autoimmune prevalence in FHHt patients was compared using the binomial test with estimates of prevalence in the general population outlined in Conrad *et al*. Numerical variables were correlated with Spearman's rank correlation with Benjamini Hochberg correction for multiple comparison testing. Analysis was performed using Graphpad PC prism version 10 (www.graphpad.com). A p-value of ≤ 0.05 was considered statistically significant.

Study approval

The Royal Free Hospital Research and Development committee approved the study (identification number 7727) and all subjects provided written informed consent prior to enrollment.

Data Availability

Values for all data points in graphs are reported in the Supporting Data Values file. Any additional relevant data are available from the corresponding author upon request.

Acknowledgements

We acknowledge the involvement of the FHHt patient cohort as research partners within this project. Moreover, we acknowledge the academic mentorship of Professor Stephen Walsh who sadly passed away during the study.

Supplementary 1: Demographic, clinical, and biochemical features of FHHt patients at the time of initial presentation to kidney services, and at the time of recruitment to the study. Figure shows calf skin apparent tissue sodium concentration measured by ^{23}Na MRI in FHHt patients (n=4) and healthy controls (n=5). Red dot represents FHHt patient not on thiazide treatment, whereas black dots in the FHHt group represent FHHt patients on thiazide treatment.

**Clinical Features at Initial Presentation**	
**Age at Diagnosis** (years) [median; inter-quartile range]	39 (24-45)
**Primary Presenting Feature** N, (%)	
Hypertension	3 (23)
Hyperkalaemia	7 (54)
Family History of FHHt	3 (23)
**Blood pressure** (mmHg) [median; inter-quartile range]	
Systolic	135 [121-152]
Diastolic	78 [72-82]
**Serum Potassium** (mmol/L) [median; inter-quartile range]	5.6 [5.2-6.1]
**Clinical Features at Recruitment**	
**Demographic**	
Age at Recruitment (years) [median; inter-quartile range]	44 (41-55)
Male sex N (%)	7 (54)
**Blood pressure** (mmHg) [median; inter-quartile range]	
Systolic sitting	125 [112-143]
Diastolic sitting	79 [72-88]
Systolic standing	123 [120-134]
Diastolic standing	83 [73-85]
Heart Rate (beats per minute) [median; inter-quartile range]	77 [72-89]
**Serum Electrolyte Concentration** [median; inter-quartile range]	
Na (mmol/L)	139 [137-141]
K (mmol/L)	4.8 [4.3-5.1]
Bicarbonate (mmol/L)	23 [21-24]
Cl (mmol/L)	102 [99-106]

Creatinine (μmol/L)<br>Adjusted Calcium (mmol/L)<br>Phosphate (mmol/L)<br>Magnesium (mmol/L)	76 [69-90]<br>2.35 [2.33-2.4]<br>1.11 [1.06-1.21]<br>0.85 [0.81-0.89]
Renin activity (nmol/L/h) [median; inter-quartile range]	1 [0.4-2.35]
Aldosterone (pmol/L) [median; inter-quartile range]	224 [85-385]
Aldosterone: renin activity [median; inter-quartile range]	267 [142-530]
**Urine Chemistry** [median; inter-quartile range]<br>Calcium:Creatinine<br>Fractional excretion of Potassium (%)<br>Fractional excretion of Chloride (%)	 0.39 [0.19-0.63]<br>18.5 [10.8-21.4]<br>1.06 [0.81-1.51]
**Anti-Hypertensive therapy** N (%)<br>Thiazide and thiazide-like diuretics<br>Amlodipine<br>Doxazosin	 8 (61.5)<br>1 (7.7)<br>1 (7.7)

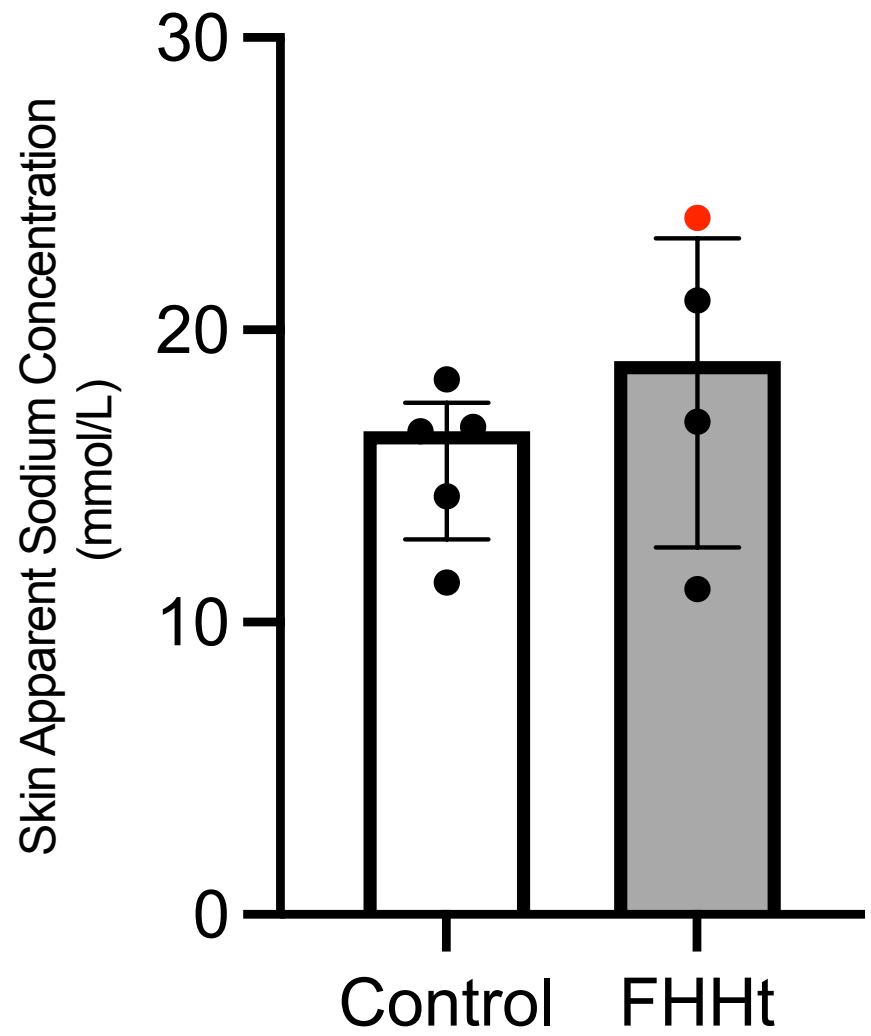

<u>Supplementary 2:</u> Clinical features of dysregulated immunity in patients with FHHt compared to healthy and disease controls, and patients with SLT. Disease controls are patients with tubular kidney disease that is not associated with a change in sodium balance. *SLT diagnoses include Bartter syndrome = 23, Gitelman syndrome = 22, and EAST syndrome = 2. ** Disease control diagnoses include proximal tubulopathy = 7, genetic distal Renal Tubular Acidosis (dRTA) = 3, tubulointerstitial nephritis (TIN) = 3, hypokalaemia due to eating disorder = 2, STX16 mutation = 2, TRMP6 Magnesium wasting = 1, Medullary Sponge Kidney = 1. Groups are compared with Fisher's exact test without adjustment for multiple comparison testing.

	Healthy Control N=16	FHHt N=13	SLT N=47	Disease controls N=19	*p*-value (FHHt vs. Healthy controls)	*p*-value (FHHt vs. SLT)	*p*-value (FHHt vs. Disease Controls)
<u>Demographics</u>							
Median Age (years) [IQR]	41 [35-55]	44 [41-55]	35 [28-43]	46 [27-52]	0.72	0.05	0.17
Male sex (%)	8 (50)	7 (54)	21 (44.7)	10 (53)	0.83	0.75	>0.99
<u>Bacterial Infections (N; %)</u>							
Abscess	0	1 (7.7)	10 (21.3)	0	>0.99	0.43	0.37
Pneumonia	0	1 (7.7)	8 (17.0)	2 (10.5)	0.45	0.67	>0.99
Recurrent or severe Upper Respiratory Tract Infection	2 (12.5)	7 (53.8)	23 (48.9)	1 (5.3)	**0.04**	>0.99	**0.0032**
Recurrent Urinary Tract Infections (UTIs)	1 (6.3)	1 (7.7)	17 (36.2)	3 (15.7)	>0.99	0.08	0.63
Other bacterial infections	1 (6.3)	0	12 (25.5)	2 (10.5)	>0.99	0.05	0.5
Fungal Infections (N;%)							
2 or more episodes of vaginal or oral Candidiasis	2 (12.5)	3 (23.1)	18 (38.3)	4 (21.1)	0.63	0.51	>0.99
Fungal Nail Infections	1 (6.3)	0	6 (12.8)	0	>0.99	0.32	>0.99
Viral Infections (N;%)							
Any recurrent or severe viral infections	3 (18.8)	9 (69.2)	14 (33.3)	3 (15.8)	**0.02**	**0.02**	**0.0035**
Recurrent Varicella Zoster Virus	2 (12.5)	1 (7.7)	1 (2.3)	0	>0.99	0.39	0.4

Recurrent Human Papilloma Virus	0	4 (30.8)	9 (21.4)	1 (5.3)	**0.03**	0.45	0.13
Recurrent Herpes Simplex Virus	1 (6.3)	4 (30.8)	6 (14.2)	0	0.144	0.20	**0.02**
Atopy (N;%)							
Any atopic features	5 (31.3)	4 (30.8)	23 (48.9)	8 (42.1)	>0.99	0.36	>0.99
Asthma	1 (6.3)	1 (7.7)	8 (17)	3 (15.8)	>0.99	0.67	0.63
Eczema/atopic dermatitis	0	1 (7.7)	15 (31.9)	3 (15.8)	0.45	0.15	0.63
Hay fever/non-allergic rhinitis	3 (18.8)	4 (30.8)	16 (34)	6 (31.6)	0.67	>0.99	>0.99
Allergy (N;%)							
Any allergies	3 (18.8)	2 (15.4)	28 (59.6)	9 (47.4)	>0.99	**0.01**	0.13
Contact/environmental allergy	1 (6.3)	1 (7.7)	17 (36.2)	3 (13.6)	>0.99	0.08	>0.99
Food Allergy	1 (6.3)	1 (7.7)	8 (17.1)	6 (27.2)	>0.99	0.67	0.22
Drug allergy	1 (6.3)	0	12 (25.5)	0	>0.99	0.05	>0.99

Supplementary 3: Initial immunological investigation of patients with FHHt. Figure demonstrates the CD4:CD8 ratio. Dotted line on the y-axis represents the upper limit of the laboratory normal range. Of the four FHHt patients with elevated CD4:CD8 ratios, two had mutations in WNK4, one in WNK1, and one in KLHL3.

	Normal Range	FHHt (Median; Interquartile range)
Haemoglobin (g/l)	110-150 (F);<br>135- 170 (M)	145 (133.5-154.5)
White Cell Count (x10^9/l)	3.5-11	6.78 (5.77-8.03)
Neutrophil (x10^9/l)	1.7-7.5	4.65 (3.95-5.20)
Lymphocyte (x10^9/l)	1-4	1.49 (1.38-2.48)
Monocyte (x10^9/l)	0.2-1.5	0.46 (0.41-0.51)
Eosinophil (x10^9/l)	0-0.5	0.11 (0.05-0.25)
Basophil (x10^9/l)	0-0.1	0.04 (0.03-0.06)
C-Reactive Protein (mg/L)	0-5	1.05 (0.83-5.4)
Immunoglobulin A (g/l)	0.7-4	2.1 (1.5-2.5)
Immunoglobulin G (g/l)	7-16	10.6 (10.2-11.5)
Immunoglobulin M (g/l)	0.4-2.3	0.9 (0.7-1.1)
Total Immunoglobulin E (KUL)	0-120	12 (9.1-95.6)
Complement C3 (g/l)	0.9-1.8	1.035 (0.93-1.21)
Complement C4 (g/l)	0.1-0.4	0.21 (0.15-0.26)
CD3 (absolute; g/l)	0.87-2.51	1.04 (0.89-1.53)
CD4 (absolute; g/l)	0.56-1.46	0.75 (0.51-1.01)
CD8 (absolute; g/l)	0.25-0.99	0.321 (0.27-0.42)
CD19 (absolute; g/l)	0.11-0.69	0.181 (0.11-0.25)
CD16, CD56 (absolute; g/l)	0.11-0.64	0.201 (0.16-0.29)
CD3 (% of lymphocytes)	57.22-86.8	73 (68-74)
CD4 (% of lymphocytes)	30.85-60.81	50 (42-53)
CD8 (% of lymphocytes)	9.8-36.47	21 (17.8-25.4)
CD19 (% of lymphocytes)	4.16-26.11	11 (9-13)
CD16, CD56 (% of lymphocytes)	5.4-24.3	14 (10.3-18.8)
CD4:CD8	0.54-2.97	2.5 (1.9-2.9)

ANA (% positive)	<1:40 titre	7.7

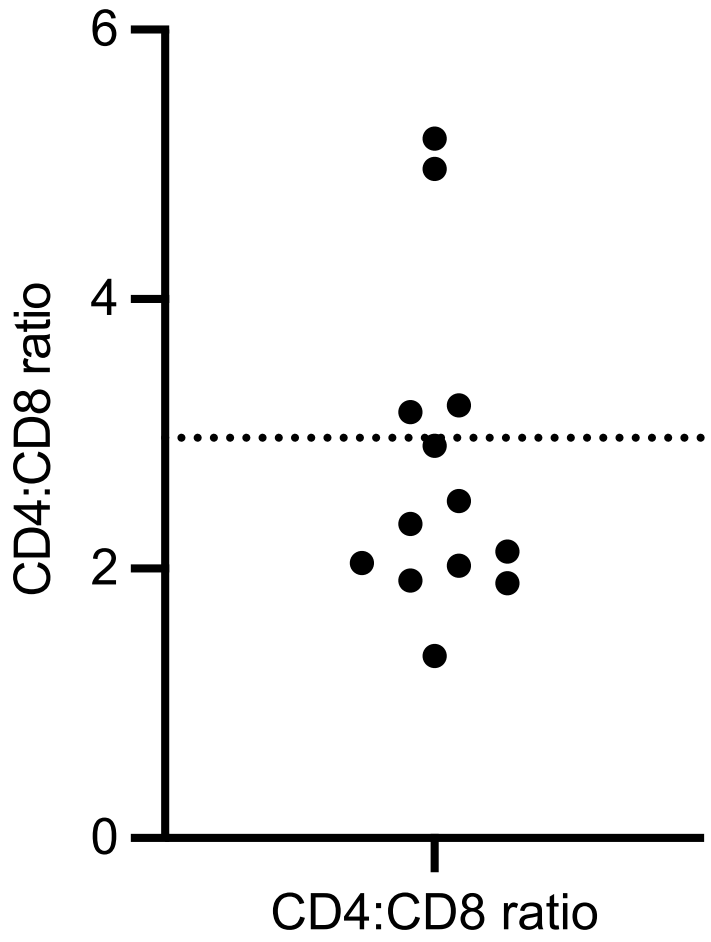

Supplementary 4: **T cell Gating Strategy**

A) Lymphocytes were gated on an FSC-SCC plot. Single cells were gated on an FSC-A FSC-H plot. B) Live cells were gated on an FSC-Viability dye plot. C) Th1 polarisation was determined by CD4 IFNγ gating. D) Th17 polarisation was determined by CD4 IL-17 gating.

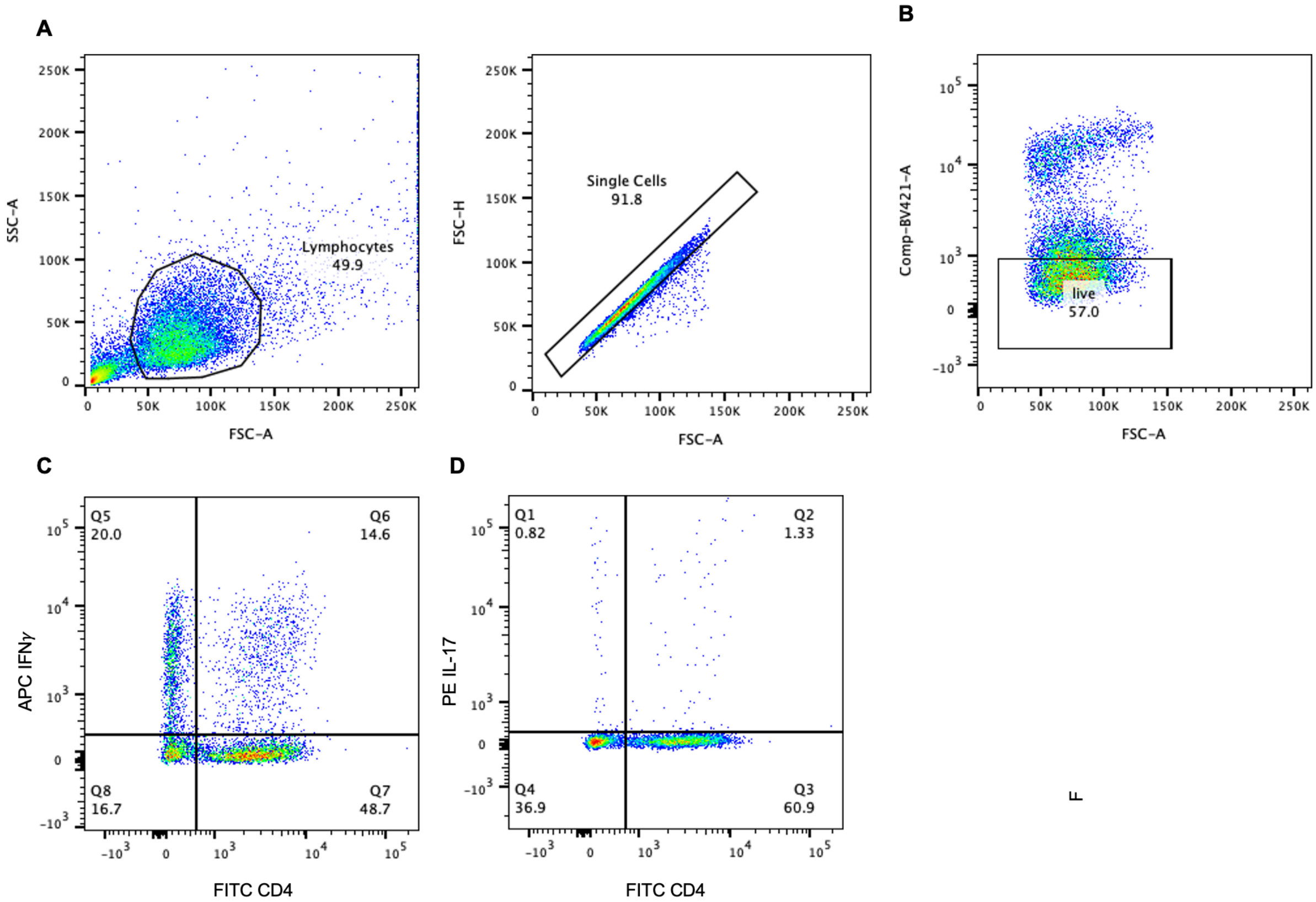

Supplement: Supplementary file 1 [file jciinsight-11-206932-s151.pdf]
